# Moisture Effects on Acoustic Emission Characteristics and Damage Mechanisms of Balsa Wood Core Composite Sandwich under 4-Point Bending

**DOI:** 10.3390/ma17051044

**Published:** 2024-02-24

**Authors:** Yuan Wu, Marianne Perrin, Marie-Laetitia Pastor, Pascal Casari, Xiaojing Gong

**Affiliations:** 1 Nantes Université, Ecole Centrale Nantes, CNRS, GeM, UMR 6183, F-44600 Saint-Nazaire, France; yuan.wu@univ-nantes.fr (Y.W.); pascal.casari@univ-nantes.fr (P.C.); 2 Institut Clément Ader (ICA), CNRS, UMR 5312, Université Toulouse III-Paul Sabatier, 1 Rue Lautréamont, 65010, Tarbes, France; marie-laetitia.pastor@iut-tarbes.fr (M.-L.P.);

**Keywords:** moisture effects, damage mechanisms, bio-based balsa wood core sandwich, acoustic emission (AE), 4-point bending tests

## Abstract

To contribute to the development of sustainable composites, this work investigates the effects of moisture on the key AE characteristics related to the damage mechanisms of a bio-based balsa wood core sandwich in 4-point bending tests, including cumulative counts, amplitude, peak frequency, and duration. Novel triple dog-bone balsa wood core sandwich specimens with different MC (moisture content) were studied by comparing microscopic observations and a proposed two-step clustering approach in AE analysis. Three MC states, i.e., dry, 50% MC, and 120% MC, are discussed. GFRP (glass-fiber-reinforced polymer) laminate skin damages were found to be predominant in most GFRP–balsa sandwich specimens, but balsa wood core damages play a more important role as MC increases. The degradation of the bending stiffness of the sandwich was proven to be faster in the first linear stage of the moisture absorption curve, while the decrease in bending strength was more pronounced at the MC saturation level. Finally, for all of the dry and wet sandwich specimens, peak frequency and duration were proven to be more helpful in identifying damages associated with the lighter bio-based balsa wood core, such as balsa core damages and skin/core debonding.

## 1. Introduction

In recent decades, natural fibers [1,2], including plant fibers, have received increasing attention in advanced industrial sectors such as aircraft and marine structures [3] owing to their light weight, strong mechanical performance, and low environmental impact. Taking into account the scientific, economic and environmental perspectives, natural fiber-based composite materials, such as balsa wood [4,5] and flax [6]-based composite sandwiches, have become good alternatives to the traditional GFRP or CFRP (glass or carbon-fiber-reinforced polymer) laminates. The design of composite sandwich structures [1,4,5] can make sure that the thin, stiff skins provide sufficient bending strength and stiffness while the lighter and thicker core carries shear stresses. Since balsa wood [7] is the lightest commercial timber in use, the desire for sustainable composite materials has driven human beings to investigate this less-known, eco-friendly wood core in depth. However, the mechanical properties of balsa wood significantly depend on density [7,8], humidity [9,10], temperature [1], etc. When balsa wood is bonded with GFRP laminate skins to manufacture an anisotropic and heterogeneous sandwich structure, the mechanical behaviors [8,11] and damage mechanisms [4,5,12,13] of the balsa core, GFRP skins, and the whole sandwich will become more complicated. Under bending loading, it has been proven that more than three damage mechanisms [4,12,13] may appear simultaneously in a sandwich structure, including core damages, skin damages, and skin/core debonding. Furthermore, damage initiation and evolution mechanisms become more complex when moisture [14,15] diffuses into the composite sandwich. To better understand the structural sustainability of a balsa wood core sandwich throughout its life cycle, the effects of moisture on its damage mechanisms should be explained more clearly.

In fact, the application of balsa wood is limited mainly due to its high hygroscopic sensibility [9,14,15]. Legrand V et al. [14] found that the saturated MC (moisture content) of pure balsa wood panels can reach 400% after being immersed in water for two months. However, regarding the balsa wood core sandwich panel, its saturated MC has only reached 80%. This means that the moisture diffusion behavior in pure balsa and a balsa wood core sandwich is different. In a sandwich, GFRP skins can protect against water spreading too fast into the balsa core. The effects of moisture on GFRP or CFRP laminates have been studied by many authors [16,17,18]. A general conclusion is that moisture diffusion will first cause internal damage in the composites by reducing the fiber/matrix interface strength. Nevertheless, for a sandwich structure, it is still unclear how moisture diffusion affects the mechanical performances of the laminate skins, the core, and skin/core interfaces at the same time. Some researchers [14,19,20,21] have demonstrated that moisture-induced stresses could occur at the skin/core interfaces in a balsa, foam, and honeycomb core sandwich, so skin/core debonding could be different from that in the dry sandwich. In 3-point bending and debonding fracture tests, Cantwell W J et al. [15,21] verified that moisture uptake would lead to a decrease in the skin/core interface strength of honeycomb/foam sandwich structures but result in an increase in GFRP–balsa sandwich. However, so far, there is no clear agreement on the influence of moisture absorption on the damage mechanisms of sandwich structures made from different core materials, especially on bio-based balsa wood core sandwiches under 4-point bending loading. Therefore, it is worth further investigating the predominant damage modes in balsa wood core sandwiches with different MC to better understand the separate role of the skin and the core.

To characterize different damage mechanisms in sandwich structures in bending tests, AE (acoustic emission) [22,23,24,25] has become a powerful tool to help identify where and when a certain kind of damage initiates and propagates. But, few references [12] can be found on the application of AE in the damage characterization of balsa wood core sandwich structures. Since the AE technique can capture the stress wave released by a growing microscopic crack [26] in a material, it is more sensitive to some microscopic damages which could not be easily detected by other NDT (nondestructive testing) methods [27,28,29,30], for example, infrared thermography [29,30]. Accordingly, AE plays a more important role in the damage classification in composite sandwich structures [12,22,24]. To classify different damage modes into separate groups by AE analysis, machine learning methods [31,32] such as K-means [22,23,24] and other UPR (unsupervised pattern recognition) clustering algorithms have been widely used. The traditional K-means algorithm has been proven to be able to classify common foam [22,23] and honeycomb [24] core damages, and skin/core debonding and laminate skin damages, but the relationship between AE characteristics and balsa wood core damages [12] is not very clear. In the clustering analysis using K-means method, the selection of the optimum number of clusters is a crucial factor that can affect the accuracy of damage classification. Different indices, such as silhouette coefficients [33], DB (Davies–Bouldin) [12,23,24], and Tou [34], have been proven to be effective for this selection in composite materials. Another important factor determining the clustering accuracy is the determination of appropriate AE parameters [22,23,24], such as amplitude, peak frequency, duration, counts, and energy. In our previous study [12], AE characteristics related to the main damage modes of common sandwich structures were concluded. We verified that the amplitude, peak frequency, and duration are the most valuable parameters for damage characterization in dry balsa wood core sandwiches at room temperature, but it is still not clear whether moisture absorption would result in a significant change in these main AE parameters. Moreover, these characteristics may be also affected by the loading condition, material properties, temperature, etc. Thus, the impact of MC on AE characteristics and the associated damage mechanisms in balsa wood core sandwiches should be explored more deeply, to lay a good foundation for the application of AE method in the field of SHM (structural health monitoring) in these bio-based composite materials in marine and other industries.

To further investigate the effects of moisture on AE parameters which carry the most relevant information about different damage modes, in this work, 4-point bending tests monitored by an AE system were performed on balsa wood core sandwich specimens containing three different MCs. All specimens have an original triple dog-bone geometry [11,12], to make it easier to observe the skin damages and core damages at different locations. All the recorded AE signals were processed by our proposed two-step clustering approach in [12], and, finally, the relationship between MC, AE characteristics, and damage mechanisms of the balsa wood core sandwich is pointed out.

## 2. Materials and Methods

### 2.1. Materials and Triple Dog-Bone Sandwich Specimens

**Figure 1:**
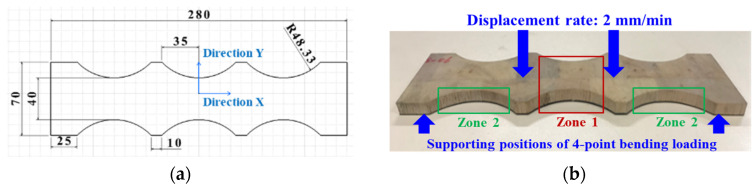
Design of a GFRP–balsa sandwich specimen. (**a**) Geometry of triple dog-bone shape; (**b**) pure bending and shear zones in the specimen.

All balsa wood core sandwich specimens are composed of two identical 3-layer ([0°]_3_) woven GFRP skins and one balsa core (Ref: BALTEK SB.100, density: 148 kg/m^3^) [11,12]. In the manufacturing process, GFRP skins and the balsa core were infused layer by layer using the resin (EPOLAM 2017) under room temperature. Additionally, then, they were cured simultaneously in the vacuum bag under room temperature for 7 h, and post-cured at 45 °C for 2 h, 60 °C for 2 h, and 80 °C for 8 h [35]. Normally, a large sandwich panel is firstly fabricated. To obtain the smaller triple dog-bone sandwich specimens, the water jet technology was used for the cutting work. This creative triple dog-bone shape (see Figure 1a) was designed to better monitor GFRP skin damages in the center pure bending zone 1 and balsa core damages in the bending and shear zone 2, considering the stress distribution [11] in a sandwich under 4-point bending loading, as seen in Figure 1b. Figure 1a shows basic dimensions (in mm) of the triple dog-bone sandwich. In addition, the average GFRP skin thickness is 0.5 mm, and the average balsa core thickness is 9 mm. Since the balsa wood fibers are along the thickness direction Z, the strength in the thickness direction of balsa wood core is much higher than that in the X–Y plane [7]. The main mechanical characteristics of the GFRP skin and balsa wood core are illustrated in Table 1 [11].

### 2.2. Preparation of Specimens with Different MC

**Figure 2:**
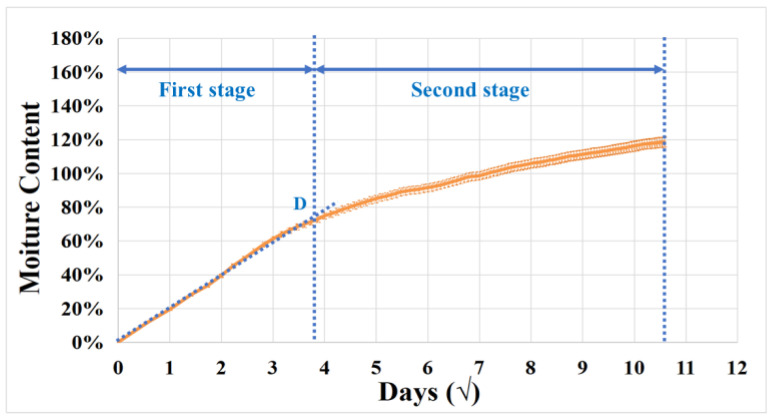
The 2-stage moisture absorption curve of a GFRP–balsa sandwich [11].

To study the moisture absorption behavior [36,37] of the balsa wood core sandwich, based on the standards ISO 12571 and ASTM D5229, five dried GFRP–balsa sandwich specimens were firstly immersed in water under room temperature until reaching the constant mass, as presented in [11]. The mass was measured every 24 h until the change between three continuous measurements was less than 0.1%. Figure 2 shows that the average moisture absorption curve reaches the transition point D after 12 days, with about 70% (±2.01%) MC, and after 112 days, the MC is nearly constant at 120% (±3.71%). It seems that this moisture diffusion process matches the dual-Fickian model [38] which can be often observed in composite materials. The overall moisture diffusion coefficient decreased in the second stage after point D, compared to the first linear stage before point D. It makes us think that the difference in moisture absorption rates between the two stages may lead to the different decrease rates of the stiffness and strength in the first and second stages. Subsequently, based on Figure 2, three wet sandwich specimens with the saturated 120% MC in the second stage were firstly tested in 4-point bending tests, to compare with the dry specimens. Next, to enrich the database of the effects of moisture on the mechanical behaviors of balsa wood core sandwiches, the moisture absorption test was repeated on the other three specimens to obtain another MC (50%) which is in the first linear stage in Figure 2. Finally, three different MCs, that is, dry, 50% MC and 120% MC, were compared in 4-point bending tests. For each MC, at least three specimens were tested under the same 4-point bending loading condition to ensure the testing accuracy.

### 2.3. 4-Point Bending Tests with AE Monitoring

**Figure 3:**
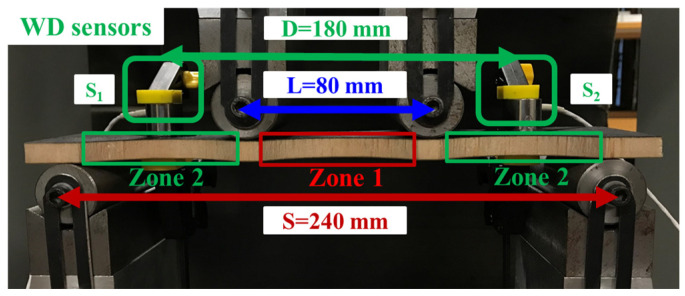
The 4-point bending test setup with AE sensor monitoring.

In 4-point bending tests, all the dry and wet specimens were tested at a displacement rate of 2 mm/min [11,12]. Figure 3 shows the support span (S = 240 mm) and loading span (L = 80 mm). The AE monitoring system is composed of two wideband sensors (100 kHz-1 MHz), two preamplifiers, analogue filters, and a PCI-2 acquisition system (Mistras AEwin for USB^TM^ software version E3.32). The distance between the two wideband sensors [12] is 180 mm. The main AE acquisition parameters [24] are concluded in Table 2. Peak definition time (PDT), hit definition time (HDT), and hit lockout time (HLT) are the most critical parameters that can make sure that the right number of hits can be obtained from the complex AE waveforms. For balsa wood core sandwiches, after repeating many trying tests, PDT, HDT, and HLT were found to be 30 μs, 100 μs, and 300 μs, respectively.

### 2.4. Two-Step Clustering Approach in AE Analysis of Sandwich Structures [12]

The clustering analysis of AE signals [22,23,24] is one of the most important issues for the damage classification in sandwich structures. In the clustering process, the selection of the suitable number of clusters and the optimum AE parameters mainly determines the final accuracy. First of all, eleven parameters [12], including energy, amplitude, duration, rise time, counts, counts to peak, average frequency, frequency centroid, peak frequency, initiation frequency, and reverberation frequency, are often chosen to recognize the possible skin and core damages. Next, to find the optimum number of clusters, three coefficients such as Davies–Bouldin (DB), Tou, and silhouette are combined [12,33,34].

**Figure 4:**
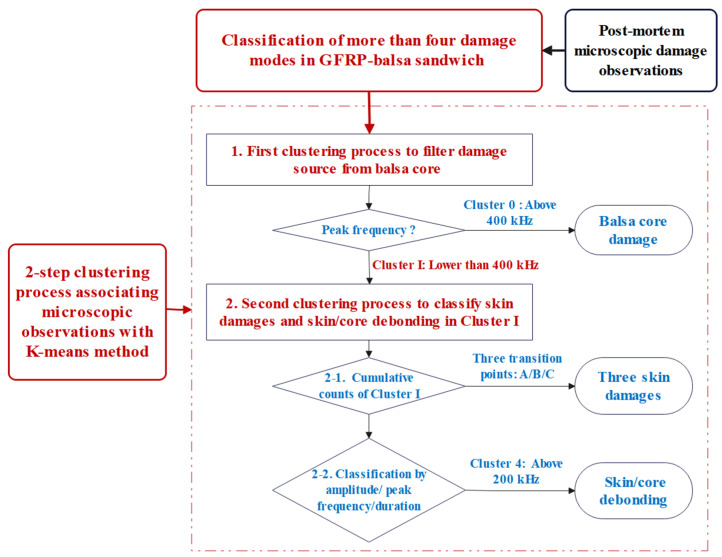
Two-step clustering approach in AE analysis of sandwich structures [12].

Normally, the number of clusters (k) is optimum when the coefficient DB is minimum, while it is better when the Tou and silhouette are higher. The detailed explanation of these three coefficients can be found in [12]. To be emphasized, all these indices may be affected by the database samples and real damage modes. Additionally, it would be interesting to prove whether these indices could be influenced by the absorbed MC. After the analysis of the optimum number of clusters, all the recorded AE signals in 4-point bending tests of dry and wet sandwich specimens can be analyzed in detail, based on our previous proposed two-step clustering approach in [12], as shown in Figure 4.

This special approach can be validated in more cases where at least two different kinds of materials exist in a sandwich. In step 1, the microscopic balsa wood core damages (Cluster 0) can be firstly identified mainly based on their higher peak frequency range. In step 2, the skin damage initiation can be identified based on the three transition points of the cumulative counts curves [12,39]. Additionally, then, skin/core debonding can be further identified mainly based on the higher peak frequency range and shorter duration.

This two-step clustering process has been proven to be effective in the dry balsa wood core sandwiches [12]. However, it is not clear whether the factor MC would affect the characteristics of these parameters. Thus, in this work, the influence of moisture absorption on AE characteristics associated with different damage modes of the GFRP–balsa sandwich will be discussed.

## 3. Moisture Effects on Damage Mechanisms of Balsa Wood Core Sandwich

### 3.1. Impact of MC on Bending Stiffness and Strength

**Figure 5:**
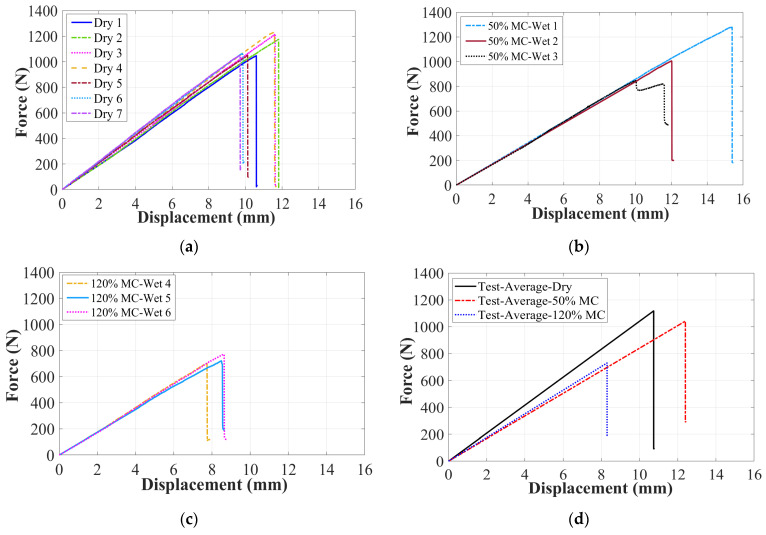
Force/displacement curves of dry and wet GFRP–balsa sandwiches. (**a**) Dry specimens; (**b**) wet specimens with 50% MC; (**c**) wet specimens with 120% MC; (**d**) average curves.

**Figure 6:**
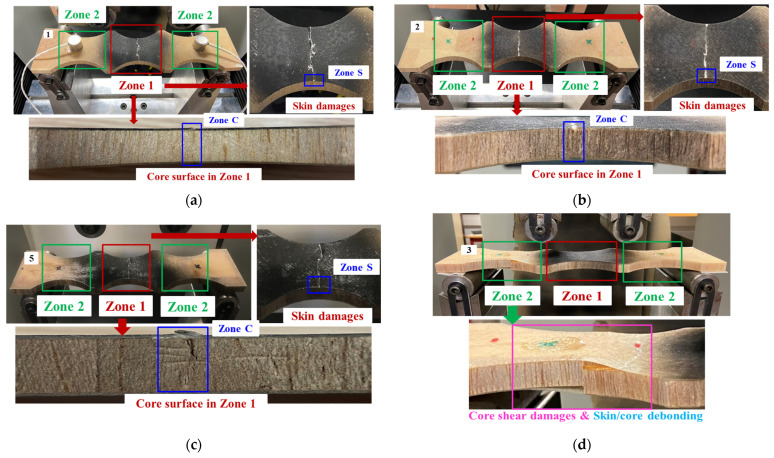
Fracture surfaces of dry and wet GFRP–balsa sandwiches. (**a**) Dry 1; (**b**) Wet 2 (50% MC); (**c**) Wet 5 (120% MC); (**d**) Wet 3 (50% MC).

Figure 5 shows force/displacement curves of all dry and wet GFRP–balsa sandwich specimens in 4-point bending tests. The quasi-linear behavior can be observed before the final fracture in most specimens except the specimen Wet 3 with 50% MC, where the load was first dropped and then raised again before the final rupture (see Figure 5b). The bending stiffness of all dry and wet specimens shows good repeatability, whereas the bending strength of all specimens shows very small dispersions, except the wet specimens with 50% MC. It indicates that moisture absorption may lead to greater randomness in the bending strength, which is related to the final fracture modes. If we inspect the fracture surfaces (see Figure 6) of all tested specimens, it is clear that the compressive GFRP laminate skin damages in the center zone 1 are dominant in all the other specimens, while only the specimen Wet 3 containing 50% MC shows the typical balsa core shear damages and skin/core debonding in the left zone 2 (see Figure 6d). This special difference in the damage modes of Wet 3 will be further explained by AE characteristics. Table 3 concludes the average values of the bending stiffness and fracture load of all dry, 50% MC, and 120% MC wet specimens.

To further investigate the impact of MC on bending stiffness and strength, as seen in Figure 5d and Table 3, the average bending stiffness of wet specimens with 50% MC and 120% MC decreased by almost 20%, compared to dry specimens. It is also interesting to find that the decrease in bending stiffness is more significant in the first faster moisture diffusion stage before point D in Figure 2. However, the average bending strength of wet specimens with 50% MC shows only a 7% decrease, while the average value of wet specimens with 120% MC has significantly decreased nearly by 35%. It means that the bending strength reduction is more pronounced at the saturation level of moisture absorption. Thus, it can be concluded that moisture absorption has less effect on the bending strength in the first quicker moisture diffusion stage in Figure 2.

Correlating this conclusion with the two-stage moisture absorption behavior in Figure 2, these phenomena should be related to the fact that the balsa wood core absorbs water faster [14] than the GFRP skin in the first stage, and MC in the balsa wood core reaches the saturation more quickly before point D. Additionally, then, in the second stage, it is the GFRP skin that mainly contributes to the moisture diffusion process in the GFRP–balsa sandwich. Since the strength of a sandwich is mainly affected by the properties of the stronger skin [11,12], the strength of the wet GFRP–balsa sandwich will decrease faster in the second stage. However, the moisture-induced thickness expansion of the wet balsa wood core could contribute more to the bending stiffness [35] degradation in the first moisture absorption stage before point D.

### 3.2. Impact of MC on Microscopic Damage Observations

**Figure 7:**
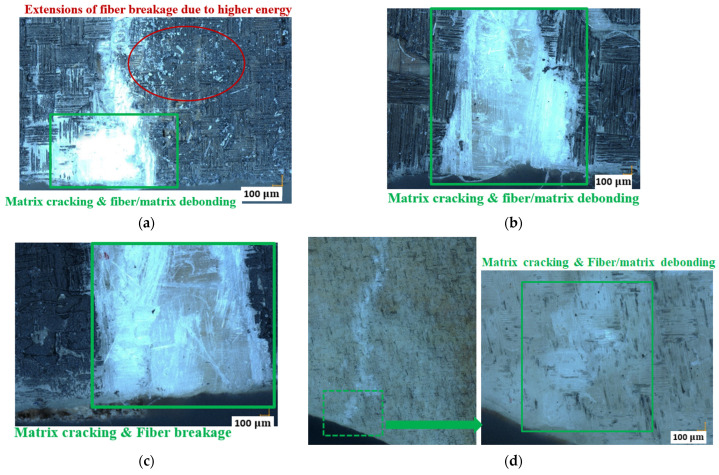
Skin damages in dry and wet specimens. (**a**) Dry 1 in zone 1; (**b**) Wet 2 (50% MC) in zone 1; (**c**) Wet 5 (120% MC) in zone 1; (**d**) Wet 3 (50% MC) in zone 2.

**Figure 8:**
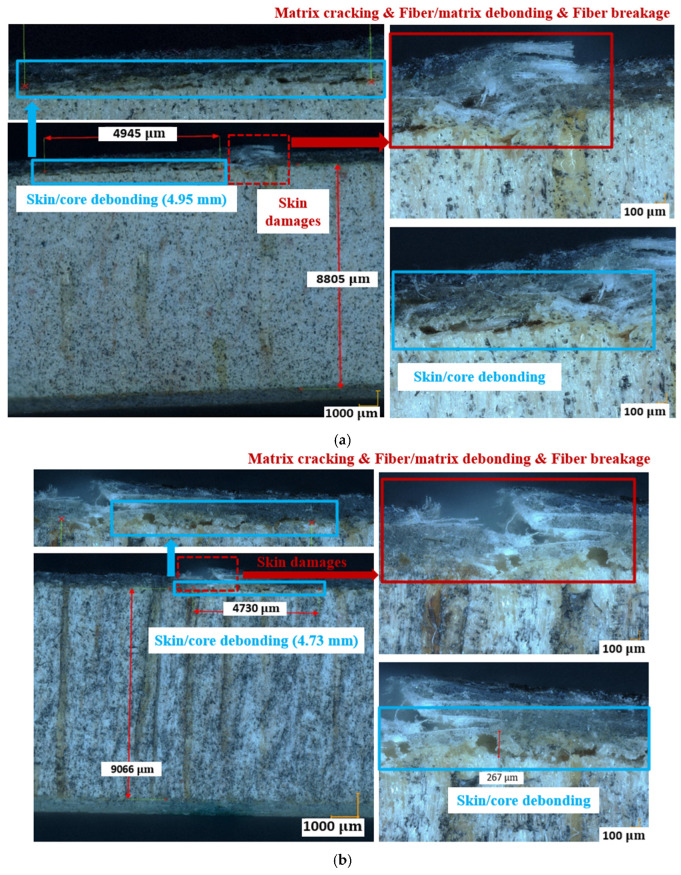

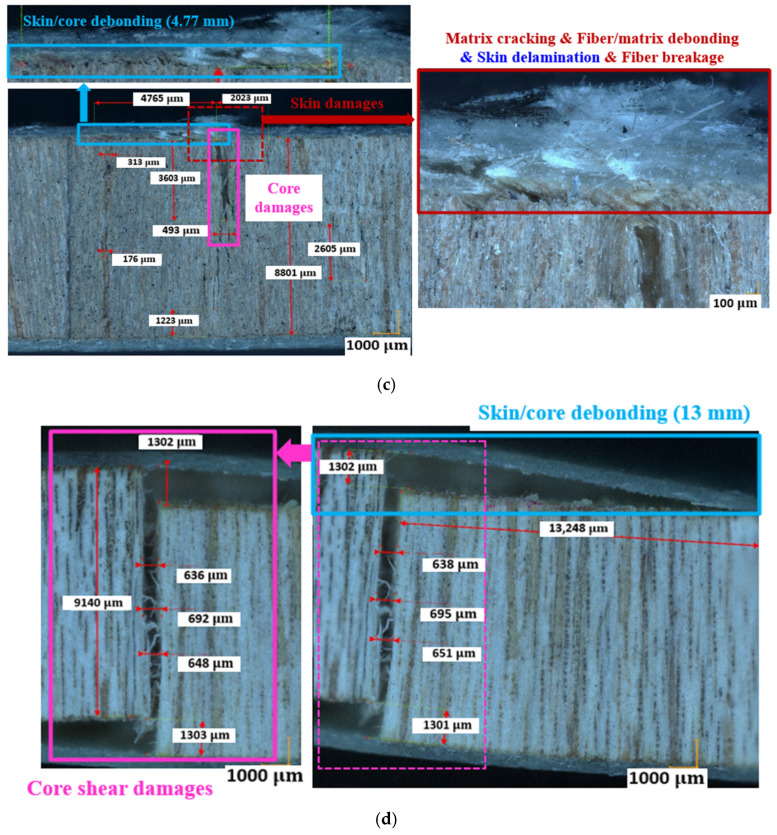
Balsa core and skin/core interface damages in dry and wet specimens. (**a**) Dry 1 in zone 1; (**b**) Wet 2 (50% MC) in zone 1; (**c**) Wet 5 (120% MC) in zone 1; (**d**) Wet 3 (50% MC) in zone 2.

To further study the complex damage mechanisms in sandwich specimens with different MC, Figure 7 and Figure 8 show microscopic images of the fractured skin (Zone S in Figure 6) and core (Zone C in Figure 6) surfaces of the four typical dry and wet sandwich specimens, observed by the microscope VHX-7000 (100X, Keyence Corporation of America, Elmwood Park, NJ, USA). Figure 7a–c compare the GFRP laminate skin damages in the center zone 1 of specimens Dry 1, Wet 2 (50% MC), and Wet 5 (120% MC). These three specimens display the similar predominant compressive skin damages in the center zone 1 at the final fracture moment. Some small extensions [11,12] appearing on the upper skin surfaces of dry specimens have proven that the dry glass fiber breakage released higher energy within a very short time, while the Wet 3 (50% MC) only shows some microscopic matrix cracking and fiber/matrix debonding in the left zone 2 of GFRP skin in Figure 7d.

Figure 8a–c show the fractured surfaces of balsa wood core and skin/core interfaces in the center zone 1 of specimens Dry 1, Wet 2 (50% MC), and Wet 5 (120% MC). Balsa wood core cracks have become more severe in zone 1 after moisture absorption. In zone 1 in specimens Dry 1, Wet 2 (50% MC), and Wet 5 (120% MC), the skin/core debonding crack lengths are 4.95 mm, 4.73 mm, and 4.77 mm, respectively. However, in Figure 8d, owing to the balsa wood core shear crack, the induced skin/core debonding crack in zone 2 of Wet 3 (50% MC) is nearly 13 mm, much longer than those in the center zone 1 of the other specimens. Therefore, after the moisture uptake, the contribution of the lighter balsa wood core to the damage mechanisms of a sandwich could become more complex and important.

Next, based on the above microscopic observations, the difference of damage mechanisms between the dry and wet specimens will be further investigated through comparing their induced AE signals.

## 4. Moisture Effects on AE Characteristics Associated with Different Damages

### 4.1. Impact of MC on AE Characteristics Related to Balsa Wood Core Damages

As explained in Figure 4, when applying the proposed two-step clustering approach in [12], the most important AE parameters related to the balsa wood core sandwich should be amplitude, peak frequency, and duration. Peak frequency is especially a key feature for the identification of lighter balsa wood core damages. However, it is still worth further demonstrating whether this two-step clustering approach can be successfully applied to the wet GFRP–balsa sandwich specimens. Hence, AE parameters of the typical three specimens Dry 1, Wet 2 (50% MC), and Wet 5 (120% MC), which show the similar predominant compressive skin damages in the center zone 1, are compared here. In addition, the special specimen Wet 3 (50% MC) will be also discussed to help verify the AE cluster associated with balsa wood core damages.

**Figure 9:**
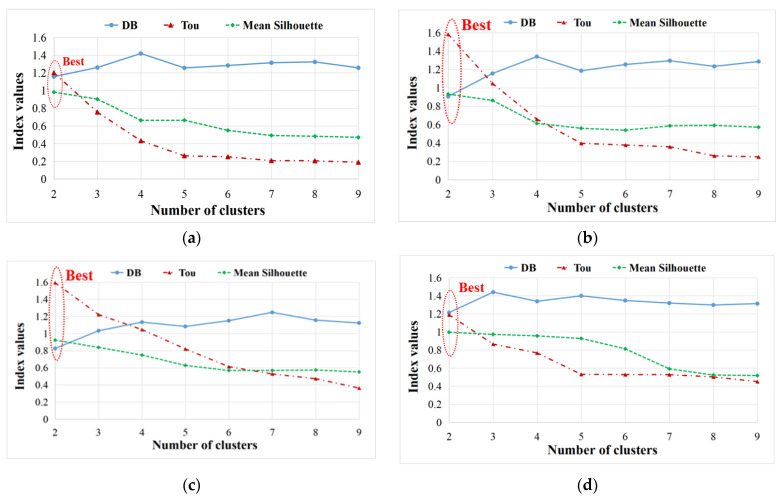
Analysis of optimum number of clusters in first clustering process. (**a**) Dry 1; (**b**) Wet 2 (50% MC); (**c**) Wet 5 (120% MC); (**d**) Wet 3 (50% MC).

In the first step of the clustering process in Figure 4, to find the optimum number of clusters, the DB/Tou/silhouette coefficients of specimens Dry 1, Wet 2 (50% MC), Wet 5 (120% MC), and Wet 3 (50% MC) are displayed in Figure 9. Obviously, for all dry and wet specimens, the optimum cluster number is 2, where DB is lowest, and Tou and silhouette are highest [12].

**Figure 10:**
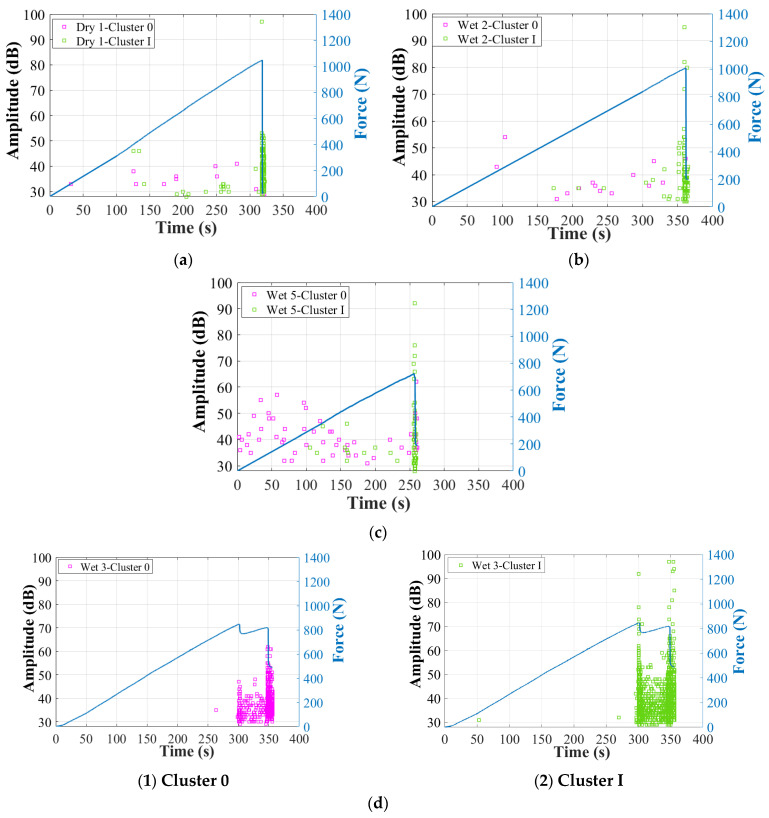
Amplitude of the two clusters after first clustering process in dry and wet GFRP–balsa sandwiches. (**a**) Dry 1; (**b**) Wet 2 (50% MC); (**c**) Wet 5 (120% MC); (**d**) Wet 3 (50% MC).

**Figure 11:**
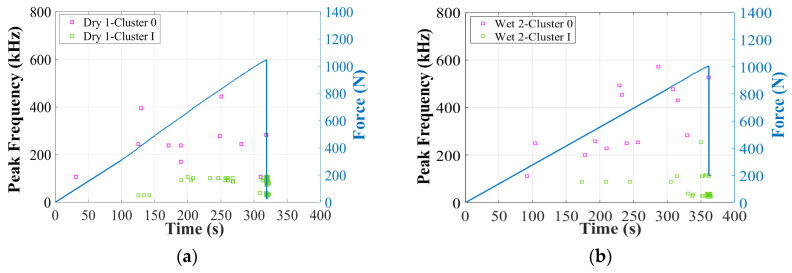

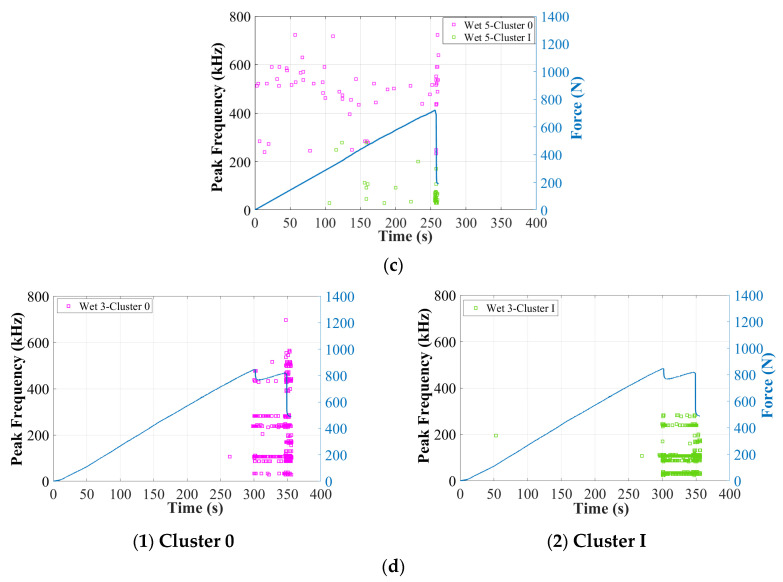
Peak frequency of the two clusters after first clustering process in dry and wet GFRP–balsa sandwiches. (**a**) Dry 1; (**b**) Wet 2 (50% MC); (**c**) Wet 5 (120% MC); (**d**) Wet 3 (50% MC).

Next, Figure 10 and Figure 11 show the correlation between amplitude, peak frequency distributions, and force/time curves. First of all, two obviously different clusters can be recognized: Cluster 0 and Cluster I. In specimens Dry 1, Wet 2 (50% MC), and Wet 5 (120% MC), AE signals higher than 60 dB (Cluster I) start to accumulate intensely just before the final fracture. Thus, Cluster I should be associated with the final dominant skin damages, with peak frequency below 400 kHz [12,39]. In the initial stage of the test, Wet 5 (120% MC) received more signals in Cluster 0, within which the amplitude is lower than 60 dB and the peak frequency is above 200 kHz. Thus, Cluster 0 should come from the balsa wood core damages [12], which are more severe in the wet specimens. In this work, particularly, as shown in Figure 10d and Figure 11d, in Wet 3 (50% MC), which presents the predominant balsa wood core shear damages in the left zone 2, the sensor S_1_ received much more AE hits in Cluster 0, compared to the other specimens. Thus, AE signals of Wet 3 (50% MC) can help verify that Cluster 0, lower than 60 dB, within a higher frequency range of 200–600 kHz, should be associated with the balsa wood core damages.

**Figure 12:**
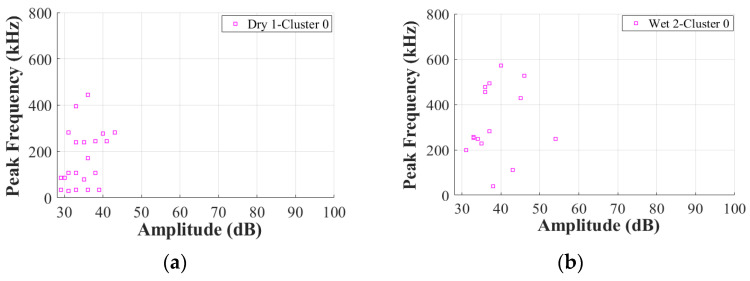

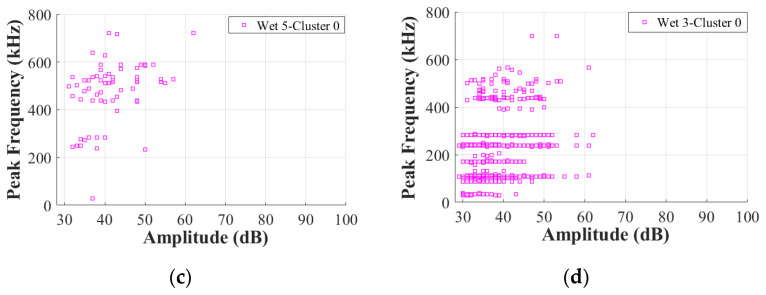
Correlation between amplitude and peak frequency in Cluster 0 in dry and wet GFRP–balsa sandwiches. (**a**) Dry 1; (**b**) Wet 2 (50% MC); (**c**) Wet 5 (120% MC); (**d**) Wet 3 (50% MC).

**Figure 13:**
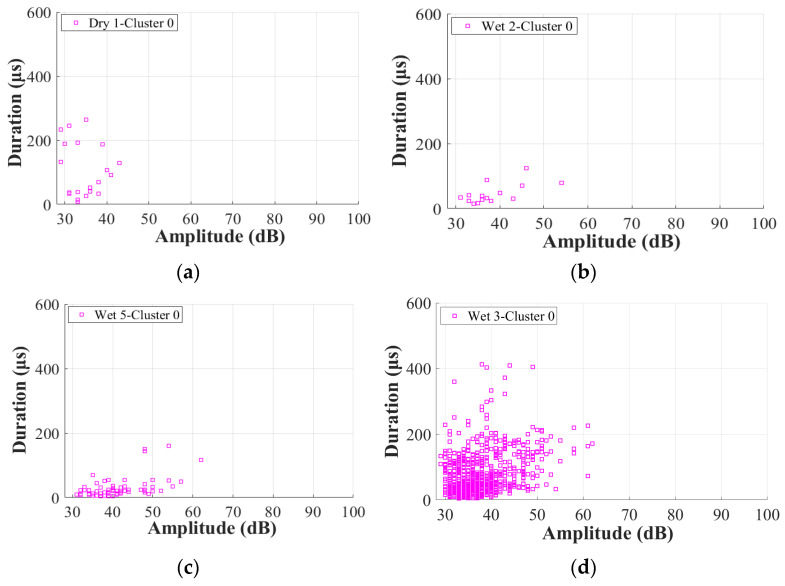
Correlation between amplitude and duration in Cluster 0 of dry and wet GFRP–balsa sandwiches. (**a**) Dry 1; (**b**) Wet 2 (50% MC); (**c**) Wet 5 (120% MC); (**d**) Wet 3 (50% MC).

As proven in [12], the duration of the balsa wood core damage is also significantly shorter than that of the GFRP skin damage. To further verify the effects of moisture on the other AE characteristics of Cluster 0 in the GFRP–balsa sandwich, Figure 12 displays the correlation between amplitude and peak frequency. Figure 13 shows the correlation between amplitude and duration. As can be observed more clearly, in Cluster 0, the amplitude is lower than 60 dB, the peak frequency is primarily below 600 kHz, and the duration is always shorter than 400 μs in all dry and wet specimens.

To conclude the impact of moisture absorption on AE characteristics, in all figures, it can be seen that more AE hits appear in Cluster 0 of the wet balsa wood core sandwich specimens. It matches well with the truth that the balsa wood core damages would become more important due to the moisture diffusion. Furthermore, it verifies that Cluster 0 can be more easily distinguished from Cluster I according to the peak frequency [12] distributions, especially after moisture absorption.

In summary, when there exist more than two different damage mechanisms, such as skin damages, core damages, and skin/core debonding, in a balsa wood core sandwich, the proposed two-step clustering process can be valid for the identification of balsa wood core damages (Cluster 0) in dry and wet specimens with different MC. After moisture absorption, the number of AE hits in Cluster 0 will be increased due to the more severe balsa wood core damages, but the primary ranges of amplitude, peak frequency, and duration of each type of damage will not vary a lot.

### 4.2. Impact of MC on AE Characteristics Related to Predominant Skin Damages

#### 4.2.1. Moisture Effects on Cumulative Counts Indicating Skin Damage Initiation

**Figure 14:**
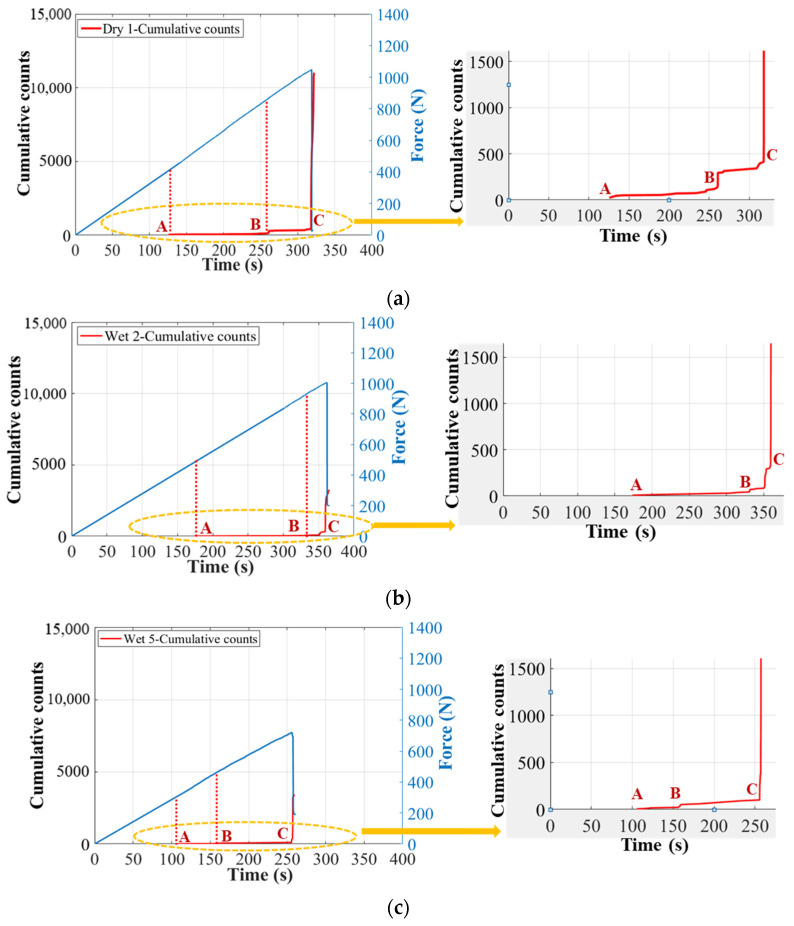
Cumulative counts vs. time in Cluster I of dry and wet GFRP–balsa sandwiches. (**a**) Dry 1; (**b**) Wet 2 (50% MC); (**c**) Wet 5 (120% MC).

Once the balsa wood core damages in Cluster 0 have been filtered in the first step of clustering process, the remaining signals in Cluster I can be further classified to associate them with the real damage mechanisms. In the second step, before the regular clustering analysis, an important parameter, the cumulative counts [39,40], should not be ignored, to help identify the skin damage initiation. The cumulative counts is a very effective indicator for identifying the crack fronts, crack openings, and crack propagation paths in composite laminates, but it has not been validated very clearly when the laminates serve as skins in composite sandwich structures [12]. Accordingly, Figure 14 plots the cumulative counts (in Cluster I) versus time in specimens Dry 1, Wet 2 (50% MC), and Wet 5 (120% MC). These three specimens are chosen here because they show the similar predominant compressive skin damages in the center zone 1 at the final fracture moment. It is interesting to find that there always exist three transition points, A, B, and C, in all cumulative counts curves. Referring to the real damage mechanisms in GFRP laminates identified by the cumulative counts in [12,39,40], point A should be the onset of the microscopic matrix cracking, point B is the start of the macroscopic fiber/matrix debonding and delamination, and point C is the beginning of the final fiber breakage.

Considering the impact of moisture absorption on the cumulative counts, point B of Wet 5 (120% MC) is advanced compared to specimens Dry 1 and Wet 2 (50% MC). It indicates that the initiation of the fiber/matrix debonding and delamination in Wet 5 (120% MC) is advanced. This is consistent with the observations in Figure 8c where the skin delamination of GFRP plies is more obvious in Wet 5 (120% MC). It is because moisture absorption degrades laminates mainly by reducing the strength of fiber/matrix interfaces [16,17,18].

Next, the effects of moisture on the three key AE parameters (amplitude, peak frequency, and duration) in Cluster I will be investigated in detail to identify the skin/core debonding from GFRP skin damages.

#### 4.2.2. Moisture Effects on AE Characteristics Related to Skin Damages

**Figure 15:**
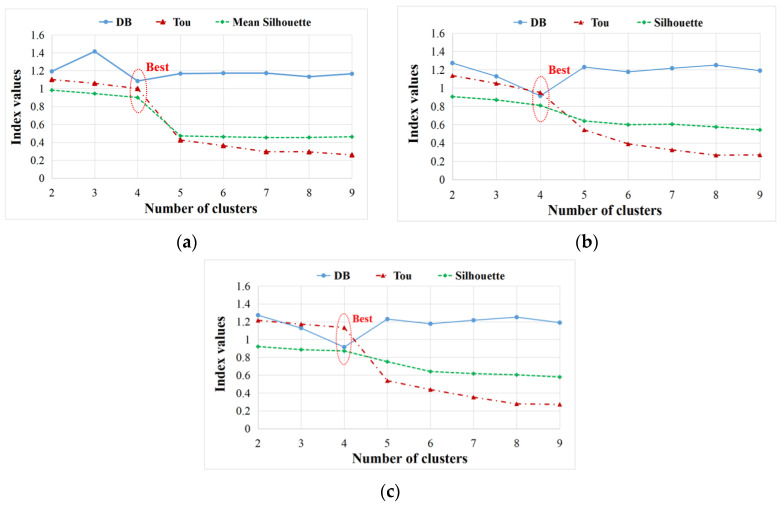
Analysis of optimum number of clusters in Cluster I. (**a**) Dry 1; (**b**) Wet 2 (50% MC); (**c**) Wet 5 (120% MC).

Like the first step in the clustering process, Figure 15 plots the coefficients DB, Tou and silhouette of Cluster I of specimens Dry 1, Wet 2 (50% MC), and Wet 5 (120% MC). It is clear that the optimum number of clusters is four for all dry and wet sandwich specimens, where DB is lowest [12,28,39]. Next, AE characteristics of the four clusters (Clusters 1, 2, 3, and 4) obtained from Cluster I will be discussed in detail.

**Figure 16:**
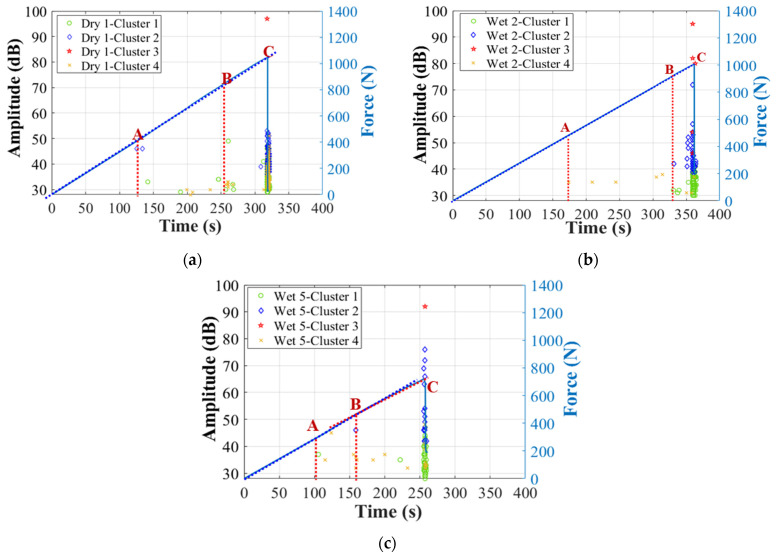
Moisture effects on AE amplitude distributions in Cluster I. (**a**) Dry 1; (**b**) Wet 2 (50% MC); (**c**) Wet 5 (120% MC).

- Moisture effects on amplitude distributions:

Figure 16 shows the difference in AE amplitude distributions of the four clusters of the three specimens with different MC. Transition points A, B, and C obtained in Figure 14 are also pointed out in Figure 16, to facilitate a better understanding of the microscopic and macroscopic damages. It is obvious that Cluster 1, Cluster 2, and Cluster 3 are the predominant damages at the final fracture moment. They should come from the GFRP skin damages. In detail, Cluster 1, lower than 50 dB, appears much earlier in the stage AB and continues to increase in the stage BC, and should be dominated by the matrix cracking and fiber/matrix debonding in GFRP laminates [12]. Cluster 2, with higher amplitude of 40–75 dB, should be skin delamination [39,40] appearing just before the final fiber breakage. Additionally, the period BC of Wet 5 (120% MC) is longer than the other specimens, indicating some small nonlinear behaviors in the end of this period of the force/time curve (see Figure 16c). It means that the skin delamination of Wet 5 (120% MC) has become more severe. The highest amplitude in Cluster 3 is above 90 dB in all dry and wet specimens, so it should be the final glass fiber breakage.

In addition to the AE characteristics, another interesting phenomenon can be observed from the number of hits of each cluster (see Table 4). Obviously, the number of hits in Cluster 4 becomes smaller in wet specimens. In all 4-point bending tests, in each specimen group with different MC, at least three experiments were repeated and the consistency was good. Hence, for each group of MC, only AE hits of a typical specimen are explained in Table 4. Table 4 also indicates that Cluster 4 should be the skin/core debonding, which is less severe in Wet 2 (50% MC) and Wet 5 (120% MC) in Figure 8 [12,15]. However, the percentage of the number of hits in Cluster 0 in wet specimens has increased a lot. It verifies that there exist more microscopic balsa core damages after MC absorption. Next, to obtain a more accurate identification of Cluster 4, which also shows a low amplitude range, the other parameters, such as peak frequency and duration, should be added to give a more convincing explanation.

**Figure 17:**
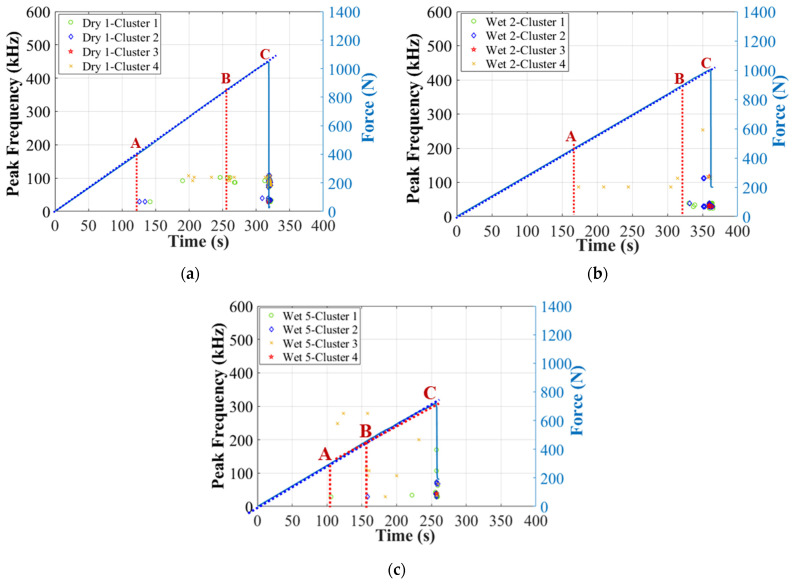
Moisture effects on AE peak frequency distributions in Cluster I. (**a**) Dry 1; (**b**) Wet 2 (50% MC); (**c**) Wet 5 (120% MC).

- Moisture effects on peak frequency distributions:

Figure 17 also displays the difference in peak frequency of the four clusters of the three specimens with different MC. Similarly, transition points A, B, and C obtained in Figure 14 are also pointed out here. It is interesting to find that most hits in Clusters 1, 2, and 3 appear within a range below 200 kHz, while Cluster 4 has more hits above 100 kHz, especially in Wet 5 (120% MC), after more moisture absorption. It demonstrates that the higher peak frequency can be an indicator to better identify the skin/core debonding. Additionally, Cluster 4 of all wet specimens shows relatively higher peak frequency than that of the dry sandwich.

**Figure 18:**
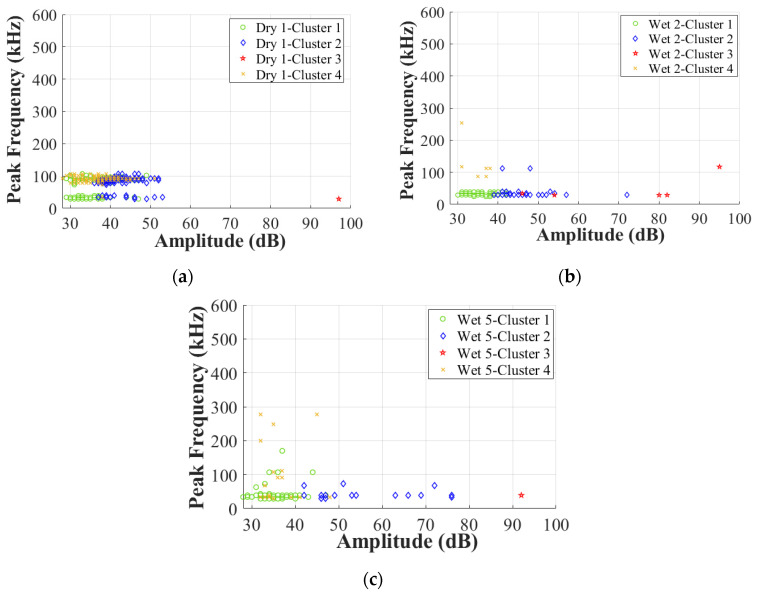
Correlation between amplitude and peak frequency in Cluster I. (**a**) Dry 1; (**b**) Wet 2 (50% MC); (**c**) Wet 5 (120% MC).

**Figure 19:**
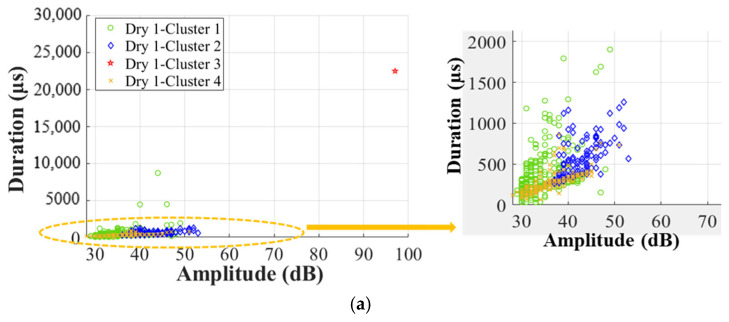

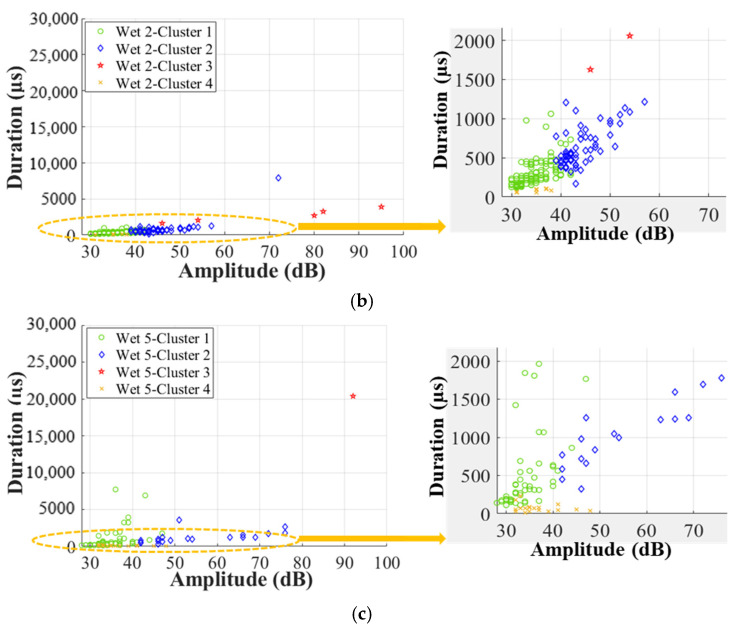
Correlation between amplitude and duration in Cluster I. (**a**) Dry 1; (**b**) Wet 2 (50% MC); (**c**) Wet 5 (120% MC).

- Moisture effects on correlations between different AE parameters:

To validate the above conclusions, Figure 18 further illustrates the correlation between amplitude and peak frequency. Figure 19 displays the correlation between amplitude and duration. As can be seen, for all dry and wet specimens, Cluster 3 shows the highest amplitude with low peak frequency below 200 kHz. Cluster 2 shows an intermediate amplitude range of 40–75 dB with low peak frequency below 200 kHz. Cluster 1 has the lowest amplitude below 50 dB with low peak frequency below 200 kHz, while Cluster 4 has a low amplitude range below 60 dB with a relatively higher peak frequency range than Clusters 1–3.

Figure 19 clearly shows that the duration in Clusters 1, 2, and 3 is longer than that in Cluster 4. Additionally, this difference is more obvious in wet specimens. In detail, the duration in Cluster 4 is shorter than 500 μs, the duration in Cluster 1 is mainly shorter than 5000 μs, and the duration in Cluster 2 is primarily shorter than 2000 μs, but Cluster 3 has the longest duration of 2000–25,000 μs. Recalling that the duration of balsa wood core damages in Cluster 0 is shorter than 400 μs, it means that the skin/core debonding and core shear damages have a shorter duration than the skin damages. This is affected by the different material properties of the skin and the core in a sandwich. It also validates that duration could be a very helpful indicator to clearly classify the skin/core debonding from various skin damages.

Finally, to further characterize the effects of moisture on different skin damages, Table 5 concludes the dominance of Cluster I without Cluster 0. It is obvious that the total number of hits in Cluster I has decreased after moisture absorption. Additionally, the percentage of Cluster 4 in Dry 1 is higher than that in the wet specimens. Correlating this with the microscopic observations in Figure 8, it can be known that Cluster 4 should be the skin/core debonding [12] which is more severe in the dry specimen [15]. However, Cluster 1 becomes more important in the wet specimens, which is related to the moisture-induced reduction of fiber/matrix interface strength [16].

### 4.3. Summary

Based on the above analysis about the impact of MC on the cumulative counts, amplitude, peak frequency, and duration, some general conclusions explaining AE characteristics related to damage modes in dry and wet GFRP–balsa sandwiches can be drawn in Table 6. However, it should be noticed that these characteristics could be affected by various factors such as the loading condition and acoustic wave propagation properties in different materials, etc. However, a meaningful conclusion is that AE characteristics of different clusters show various ranges, but each parameter of each cluster remains almost within the same range after moisture absorption. It means that our proposed two-step clustering analysis approach is valid for the balsa wood core sandwich specimens with different MC.

## 5. Conclusions

In this work, the effects of moisture absorption on AE characteristics and damage mechanisms of the bio-based balsa wood core sandwich were characterized in 4-point bending tests, based on a new two-step clustering approach. The main useful conclusions include the following:

- We demonstrate that the proposed two-step clustering approach can be valid for balsa wood core sandwich specimens with different MC. After moisture absorption, AE characteristics, including the cumulative counts, amplitude, peak frequency, and duration, can still be helpful indicators to clearly classify different balsa wood core damages, skin/core debonding, and skin damages. Among all AE parameters, peak frequency and duration are especially important for the identification of damage modes related to the lighter balsa wood core material.
- When MC increases in a sandwich, the percentage of the number of AE hits in Cluster 0 (balsa wood core damages) and Cluster 1 (matrix cracking and fiber/matrix debonding) increased, while the percentage of the number of AE hits in Cluster 4 (skin/core debonding) decreased. This helps demonstrate that moisture absorption accelerated the balsa wood core damages, matrix cracking, fiber/matrix debonding, and GFRP skin delamination, but slowed down the skin/core debonding in a GFRP–balsa sandwich.
- As concluded in Table 6, in all dry and wet balsa wood core sandwich specimens, AE characteristics of different clusters show various ranges, which can be correlated to different damage mechanisms, but the main range of each cluster related to a certain damage does not change much as MC varies.
- Considering moisture effects on 4-point bending behaviors, the degradation of bending stiffness of the balsa wood core sandwich is faster in the first quicker hygroscopic stage before point D in Figure 2, while the bending strength shows a more significant decrease mainly in the second slower moisture absorption stage. It verifies that the balsa wood core plays a more important role in the early stage of the moisture diffusion process.

In the end, considering the deeper exploration in this field, it should be first emphasized that more tests should be performed on sandwich specimens with much more MC intervals such as 25% MC, 75% MC, and 100% MC in the future. Furthermore, based on all the experimental results, numerical models can be further developed to predict moisture effects on bending damage mechanisms of balsa wood core sandwiches with different MC, by implementing the corrected mechanical parameters of the skin and the core. This correction of material parameters could be accomplished by taking into account the relationship between the variation of elastic modulus and strength of the skin and core materials, the characteristics of AE parameters, and change of MC. 

Finally, to anticipate the possible practical application of this work, firstly, the verified two-step AE clustering approach can be applied to other lightweight sandwich materials to characterize the impact of MC on defects in the marine and aeronautical structures. Moreover, the concluded AE characteristics associated with each damage mode can provide guidance for engineers and researchers to quickly recognize the different skin and core damages even in humid environments in service. In this way, the severity of the damage in a sandwich structure can be accurately assessed so that the subsequent maintenance and repair work can be carried out more effectively.

## Figures and Tables

**Table 1:** Mechanical characteristics of skin and core materials in GFRP–balsa sandwich [11].

Materials	E_1_ (GPa)	E_2_ (GPa)	G_12_ (GPa)	G_13_ (GPa)	G_23_ (GPa)	$\vartheta_{12}$
Balsa wood	0.092	0.092	0.003	0.1	0.1	0.6
GFRP	20	20	2.85	2.30	2.30	0.13

**Table 2:** AE acquisition parameters applied to GFRP–balsa sandwich [12].

Threshold (dB)	Preamplifier (dB)	Analog Filter (MHz)	PDT (μs)	HDT (μs)	HLT (μs)	Sample Rate (MSPS)	Pretrigger (μs)
28	40	0.02–3	30	100	300	5	50

**Table 3:** Average bending stiffness and fracture load of GFRP–balsa sandwich with different MC.

Specimens	Bending Stiffness (N/mm)	Standard Deviation (N/mm)	Fracture Load (N)	Standard Deviation (N)
Dry	104	4	1118	85
50% MC	84	1	1042	218
120% MC	88	3	730	37

**Table 4:** Dominance of different clusters of all damages in dry and wet GFRP–balsa sandwiches.

Specimen	Total	Cluster 0	Cluster I			
			Cluster 1	Cluster 2	Cluster 3	Cluster 4
		Microscopic Balsa Core Cracks and Interfacial Debonding	Matrix Cracking and Fiber/Matrix Debonding	Skin Delamination	Fiber Breakage	Skin/Core Debonding
**Dry 1**	%: 100	4.1	42.9	20.8	0.2	32.0
	Hits: 515	21	221	107	1	165
**Wet 2** **(50% MC)**	%: 100	7.8	54.4	31.1	2.6	4.1
	Hits: 193	15	105	60	5	8
**Wet 5** **(120% MC)**	%: 100	41.6	33.8	12.3	0.6	11.7
	Hits: 154	64	52	19	1	18

**Table 5:** Dominance of different clusters of skin damages in dry and wet GFRP–balsa sandwiches.

Specimen	Total	Cluster 1	Cluster 2	Cluster 3	Cluster 4
		Matrix Cracking and Fiber/Matrix Debonding	Skin Delamination	Fiber Breakage	Skin/Core Debonding
**Dry 1**	%: 100	44.7	21.7	0.2	33.4
	Hits: 494	221	107	1	165
**Wet 2** **(50% MC)**	%: 100	59.0	33.7	2.8	4.5
	Hits: 178	105	60	5	8
**Wet 5** **(120% MC)**	%: 100	57.8	21.1	1.1	20
	Hits: 90	52	19	1	18

**Table 6:** AE characteristics of all clusters in dry and wet GFRP–balsa sandwiches [12].

AE Parameters	Cluster 0	Cluster I			
		Cluster 1	Cluster 2	Cluster 3	Cluster 4
	Microscopic Balsa Core Cracks and Interfacial Debonding	Matrix Cracking and Fiber/Matrix Debonding	Skin Delamination	Fiber Breakage	Skin/Core Debonding
**Amplitude** **(dB)**	<60	<50	40–75	>75	<50
**Duration** **(μs)**	<400	<5000	<2000	2000–25,000	<500
**Peak Frequency (kHz)**	200–600	20–200	20–200	20–200	80–300

## Data Availability

Data are contained within the article.
